# Direct Coupling of Bio-SPME to Liquid Electron Ionization-MS/MS via a Modified Microfluidic Open Interface

**DOI:** 10.1021/jasms.0c00303

**Published:** 2020-11-20

**Authors:** Priscilla Rocío-Bautista, Giorgio Famiglini, Veronica Termopoli, Pierangela Palma, Emir Nazdrajić, Janusz Pawliszyn, Achille Cappiello

**Affiliations:** † Department of Chemistry, Life Sciences and Environmental Sustainability, University of Parma, 43121 Parma, Italy; ‡ Department of Pure and Applied Sciences, University of Urbino, 61029 Urbino, Italy; § Department of Chemistry, University of Waterloo, Waterloo, ON N2L 3G1, Canada; ∥ Chemistry Department, Vancouver Island University VIU, Nanaimo, BC V9R5S5 Canada

**Keywords:** electron ionization, liquid−EI interface, LEI, SPME, microfluidic open interface, MOI, fentanyl, matrix effects, nano-LC-MS/MS

## Abstract

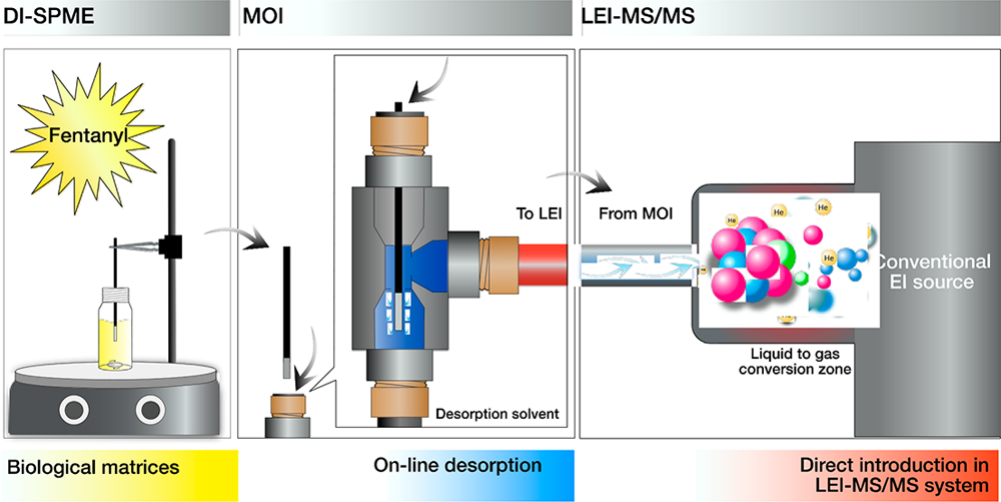

We present a modified microfluidic open interface (MOI) for the direct coupling of Bio-SPME to a liquid electron ionization-tandem mass spectrometry (LEI-MS/MS) system as a sensitive technique that can directly analyze biological samples without the need for sample cleanup or chromatographic separations as well as without measurable matrix effects (ME). We selected fentanyl as test compound. The method uses a C18 Bio-SPME fiber by direct immersion (DI) in urine and plasma and the subsequent quick desorption (1 min) in a flow-isolated volume (2.5 μL) filled with an internal standard–acetonitrile solution. The sample is then transferred to an EI source of a triple-quadrupole mass spectrometer via a LEI interface at a nanoscale flow rate. The desorption and analysis procedure requires less than 10 min. Up to 150 samples can be analyzed without observing a performance decline, with fentanyl quantitation at microgram-per-liter levels. The method workflow is extremely dependable, relatively fast, sustainable, and leads to reproducible results that enable the high-throughput screening of various biological samples.

## Introduction

Fentanyl and its derivatives have been quantified in biological fluids with GC-MS or LC-MS.^1−8^ In LC-MS/MS, internal standards are used to assess matrix effects (ME) coming from the ionization step. The chromatographic column provides the required resolution and sensitivity for analyte separation, with limits of detection below 1 ng·mL^–1^. However, LC can slow down the analytical process, especially when a high throughput is required.^9−11^ Direct methods are now widely used for the rapid screening of drugs of abuse in biofluids. Recently, Vandergrift et al. proposed a method based on paper spray mass spectrometry for the semiquantitative measurement of fentanyl and norfentanyl in urine and analgesic slurries, demonstrating the advantage of this approach in terms of sensitivity, selectivity, and rapidity for the direct sampling and prescreening of opioids.^12^

In forensic laboratories, microextraction methods are widely used as valid alternatives to conventional solid-phase, liquid–liquid, supercritical fluid, and other classical extraction methods. Microextraction strategies, regardless of if they are based on sorbent or solvent, are centered on the concept of “green chemistry” using a minimal solvent volume, thus limiting the environmental impact. Among them, solid-phase microextraction (SPME) is a well-established sampling technique, which has been broadly investigated in various application fields since its first introduction in 1990.^13−18^ A recent review summarizes new developments in that technology, showing its versality for coupling with different analytical instrumentations and expanding the range of applications.^19^ For example, Gorynski explored the role of SPME in drugs of abuse and antidoping applications, describing the different extraction modes, geometries, sorbents, and configurations compatible with GC- and LC-MS instruments.^20^

LC-MS instruments available on the market are equipped with atmospheric-pressure ionization (API) techniques, such as electrospray ionization (ESI), atmospheric-pressure chemical ionization (APCI), and atmospheric-pressure photoionization (APPI). These are all soft ionization techniques, producing protonated or deprotonated molecules (with or without adducts). The careful identification and quantification of the analytes is possible only with MS/MS or high-resolution MS (HRMS). Among them, ESI-based platforms are the most diffused for their robustness, sensitivity, and extended molecular weight range. Electron ionization (EI) is the ionization technique classically used in gas chromatography–mass spectrometry (GC-MS); however, it has been successfully applied to LC-MS analysis. Different EI sources for LC-MS have been described using different approaches.^21−26^ Cappiello’s research group recently developed the liquid electron ionization (LEI) LC-MS interface.^27−31^ LEI excels at nanoflow rates^28^ where the analytes vaporize at atmospheric pressure inside a specific vaporization microchannel (VMC) before reaching the ion source. Once in the ion source, the analytes are ionized under the typical EI conditions (70 eV), generating high-quality and library-searchable EI spectra.^28−30^

SPME, coupled directly to a MS, has recently become more popular. Besides avoiding lengthy chromatographic separations, the greatest advantage lies in using small desorption volumes that are directly introduced to the MS, thus yielding intense analytical signals. In this line, SPME has been coupled to different ionization techniques, becoming an effective means for the quantitative determination of a large number of analytes in a wide variety of application fields for the rapid quantitation and screening of a broad range of compounds present in different matrices.^31^ Many such couplings include ESI as the ionization mechanism. Methods using ESI tend to be susceptible to ME that cause signal suppression or enhancement due to high mass flow and the coelution of other compounds present in the matrix.^32,33^ Sometimes, mobile phase additives or analyte derivatives may influence ionization mechanisms.^34−36^ ME are typically evaluated using post-extraction addition or post-column infusion methods.^37−39^ Unlike ESI, EI involves a direct 70 eV interaction under vacuum, significantly reducing the effects of the matrix. The advantages of EI were successfully applied in a nano-LC-EIMS field-portable instrument for the analysis of illicit drugs.^40^ It can be used as an alternative to directly couple SPME devices to a MS via the appropriate interfaces. In this work, MOI was suitably modified to allow the direct desorption of Bio-SPME fibers coupled to a LEI-MS/MS instrument. The internal volume of the MOI was redesigned to be compatible with the nanoflow requirements needed for the proper use of LEI-MS/MS. The absence of matrix effects is a point of strength of LEI interface, as demonstrated also in this case for the matrices investigated (urine and plasma). This characteristic is particularly advantageous when no chromatographic separation is involved, fully exploiting MS/MS selectivity without affecting the quality of the quantitative data and permitting a high-throughput analysis. The determination of undiluted samples was carried out, allowing a more realistic view of the possible ME. Furthermore, the complete method was performed at nanoflow rates, which implies a minimum consumption of organic solvents.

## Experimental Section

### Materials and Supplies

Fentanyl (CAS no. 437-38-7) and fentanyl-D5 (CAS no. 118357-29-2) standard solutions were provided by Cerilliant, Sigma-Aldrich (Milan, Italy) at the concentration of 100 mg·L^–1^ in methanol (MeOH). Working standard solutions of fentanyl were volumetrically prepared daily in water or urine (50, 100, 200, and 2000 μg·L^–1^ for protocol optimization and 10, 50, 100, 200, 500, 750, and 1000 μg·L^–1^ for calibration curves). Fentanyl-D5 was used as the internal standard (IS) at the concentration of 2 mg·L^–1^ in acetonitrile (ACN). For DI-SPME optimization and calibration, the working solutions were prepared in ultrapure water with 0.5% MeOH (v/v) and in plasma and urine with 5% MeOH (v/v). All solutions were stored at 4 °C in dark vials (Agilent Technologies, Santa Clara, CA). A 250 μL Hamilton syringe provided by Merck (Darmstadt, Germany) was used to introduce the IS in the injector loop. LC-grade solvents, including ACN, ultrapure water, isopropanol, and MeOH, were purchased from VWR International (Milan, Italy).

DI-SPME studies were carried out in 4 mL glass vials with septum caps supplied by Agilent Technologies. Polyacrylonitrile (PAN) particles were used as precursors to coat nitinol wires (200 μm diameter) to obtain Bio-SPME fibers. These fibers were used for the extraction procedure. The fibers were obtained as described in a previous article.^41^ All manufactured fibers were 1 cm long and of an approximately 20 μm coating thickness. Human urine and plasma samples were collected from a healthy volunteer. Urine was collected in the morning (first urine of the day). Plasma was collected at Urbino’s hospital facility. The sample filtration of biofluids was not needed because PAN as binder has the ability to repel hydrophobic groups, thus minimizing matrix precipitation onto the coating surface. A wash step after extraction removes any loosely attached matrix components. Biological samples were protected from light and stored at 4 °C in the laboratory refrigerator.

### Instruments and Equipment

**Figure 1:**
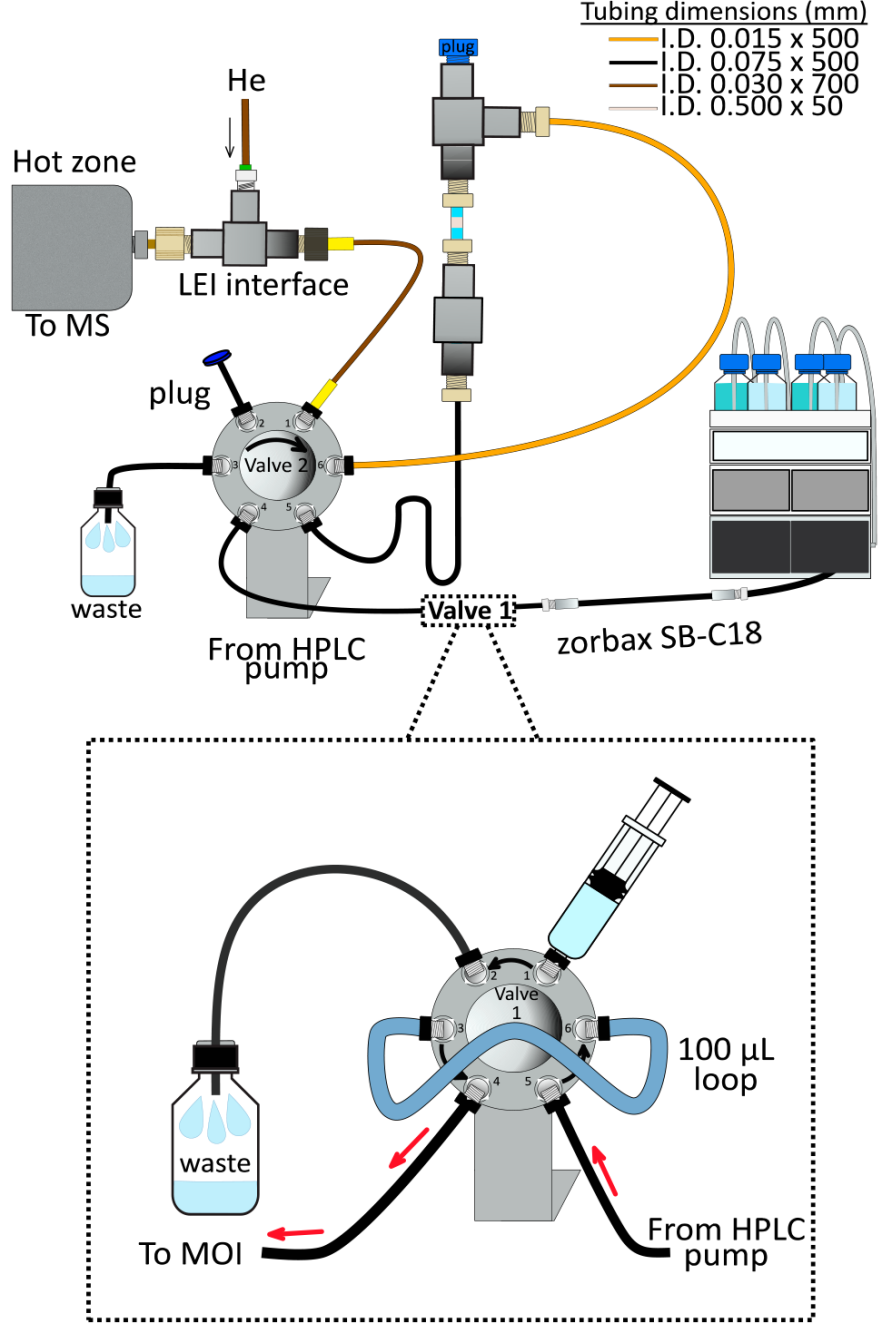
Schematics of the MOI-LEI-MS system.

The instrumentation used is shown in Figure 1 and consists of a binary nano-LC pump (Agilent 1100), a GC (Agilent 7890B), a MS detector (Agilent 7010 QqQ triple quadrupole MS equipped with a high-efficiency source (HES)), and a LEI interface. An Agilent Zorbax 300-SB C18 back pressure column (0.1 × 150 × 3.5 μm) was employed to stabilize the nanoflow rate. During the procedure, 100% ACN circulated through the system at a 400 nL·min^–1^ flow rate. A six-port valve (Agilent Valve Kit 5067-42412 ultrahigh-pressure valve head; valve 1) with a 100 μL loop was situated after the nano-LC and used to infuse the IS in the system. Another six-port valve (valve 2) was used to connect valve 1, the MOI, and the LEI-MS/MS (Cheminert, VICI, Schenkon, Switzerland). The MS ion source was kept at 280 °C. Data acquisition was carried out in multiple reaction monitoring (MRM) using the following transitions and collision energies: fentanyl, *Q* = 245–189 (10 eV) and *q* = 245–146 (5 eV); and fentanyl-D5 (IS), *Q* = 250–194 (10 eV) and *q* = 250–151 (10 eV) as shown in Table S1. Full scan analyses were conducted in an *m*/*z* range of 80–340 with a 700 ms scan time, 1.4 cycles per second, and threshold 10.

### The LEI Interface

LEI efficiently converts a liquid effluent to a gas-phase mixture of solutes and mobile phase solvents addressed to a conventional EI source. The vaporization takes place inside a long and narrow tubing (800 μm o.d., 400 μm i.d.) called a vaporization microchannel (VMC). The VMC was kept at 400 °C for all experiments. A 150 μm o.d. and 30 μm i.d. capillary tubing delivers the liquid sample inside the VMC. A coaxial He flow (1 mL·min^–1^) helps the quick transfer of the vapors, reducing the chances of thermal decomposition and preserving the original sample composition. The GC controls the VMC temperature and the He flow rate. A detailed description of LEI is available in the literature.^28,30^

### MOI Description, Modification, And Operation

**Figure 2:**
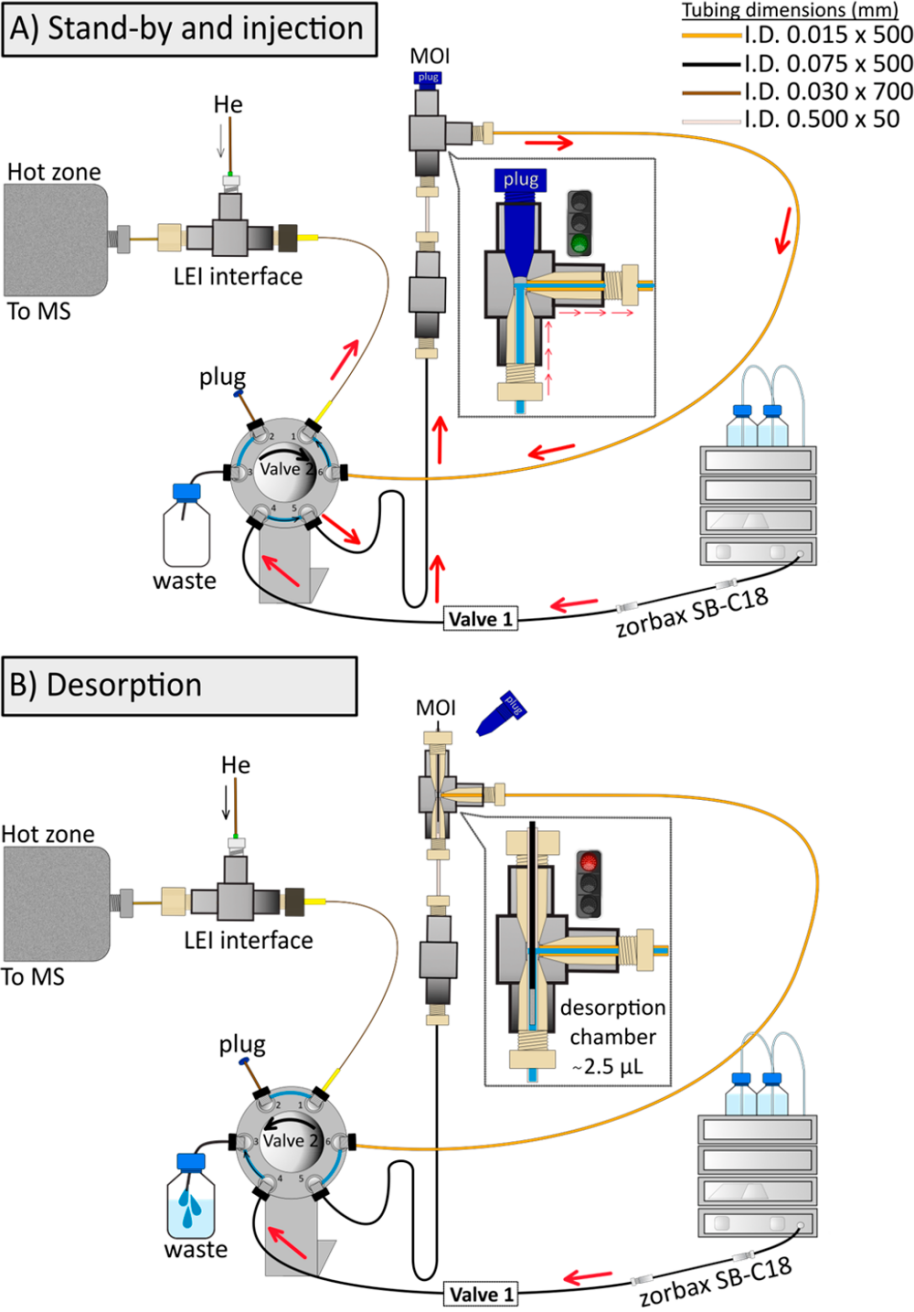
Schematics of the hydrodynamics of the MOI-LEI-MS/MS system. (A) Standby and injection position. (B) Desorption position.

The original MOI design was conceived to work at microliters-per-minute flow rates, ensuring a quick transfer of the desorbed analytes to the MS.^40^ LEI requires nanoscale flow rates, so the MOI needed a substantial modification in terms of internal volumes and connections. The use of a nano-LC system involves zero-dead volume connections, low flow rates, and high pressures. This scaled-down system implies a radical change in the desorption chamber design to reduce the flow-isolated volume to a minimum. To create a flow isolated volume of 9.8 μL, 5 cm of 500 μm i.d. PEEK tubing was used. The fiber entrance of the flow-isolated volume is normally closed by a removable plug. The plug is temporarily removed only when the fiber is inserted for the desorption step. Considering that the sorbent phase of the SPME fiber is 1 cm long, once the fiber is inside the chamber it reduces the desorption volume surrounding the coating at approximately 2.5 μL. This generates a sample peak a few minutes wide (at a 400 nL·min^–1^ flow rate) in the MS. Smaller volumes cannot be used due to the restriction caused by the fiber diameter (240 μm) and connections. A scheme of the modified version of the MOI-LEI-MS/MS system is shown in Figure 2 A and B, which reports all types and dimensions of the capillaries used in the system (IDEX, Oak Harbor, WA). The overall procedure includes the following steps:

- *Step 1: MOI filling (Figure 2A)*. A 100 μL sample loop in valve 1 was manually filled with the internal standard solution (IS, fentanyl-D5 at 2 mg·L^–1^ in ACN). A nanopump provided a flow rate of 100% ACN at 400 nL·min^–1^. After loop filling, valve 1 was switched to position A (injection). The ACN flow rate pushed the loop content to valve 2. Valve 2, placed after valve 1, worked as a bypass valve and was kept in position A (nonbypass) during this step. In this way, the IS-ACN solution coming from the loop first filled the MOI and was then directed to the LEI-MS/MS system.
- *Step 2: Fiber desorption (Figure 2B).* During desorption, the IS-ACN solution in the MOI must be isolated from the system. Valve 2 was switched to position B (bypass), and the flow was directed to waste. The plug was removed, and the fiber was inserted into the MOI for 1 min.
- *Step 3: Analysis.* After 1 min, the fiber was removed, and the plug was inserted again. Valve 2 was switched to position A (nonbypass). The IS-ACN solution containing the desorbed fentanyl was allowed to move from the MOI to the LEI-MS/MS.

### DI-SPME MOI-LEI-MS/MS System

The performance of DI-SPME-MOI-LEI-MS/MS was evaluated in water and urine. Plasma was used for the matrix effects evaluation only. The extraction conditions were optimized in water and urine. In the case of plasma, the same conditions used for urine were adopted after a dilution with water. The extraction experiments in water permitted us to optimize the MOI-LEI-MS/MS system response and configuration. The experiments were carried out in 4 mL glass vials containing a magnetic stir bar. During extraction, the Bio-SPME fiber was directly exposed to 3 mL of a liquid solution containing a known amount of fentanyl (the concentration depended on the specific experiment). The optimized conditions are as follows:

- *Water.* An aqueous standard solution of fentanyl (3 mL) containing 0.5% MeOH (v/v) at a neutral pH was subjected to 700 rpm magnetic stirring for 60 min at room temperature. Afterward, the fiber was desorbed in the MOI for 1 min.
- *Urine.* Urine (3 mL) was spiked with a known concentration of fentanyl and basified at pH 10 using NaOH (5 mol·L^–1^). After centrifugation at 5000 rpm for 5 min, 5% (v/v) MeOH was added. The samples were extracted at 700 rpm for 30 min. Then, the fiber was desorbed in the MOI for 1 min.
- *Plasma.* Plasma (1.5 mL) was diluted 1:1 (v/v) with deionized water. The procedure was the same as that employed for the urine samples (pH 10, 5% MeOH).

The desorption was carried out as described in the previous paragraph. After desorption, the fiber was immersed in isopropanol for 15 min for cleaning and conditioning before further sampling. The half-life of the fiber was estimated to be approximately 150 cycles for all samples.

## Results and Discussion

### Optimization of MOI-LEI-MS/MS

To obtain the best signal in terms of the peak shape and signal-to-noise ratio, the i.d. and position of silica inlet capillary were studied. In these studies, fentanyl was injected in the flow injection analysis (FIA) mode at the concentration of 100 mg·L^–1^ in MeOH using a 10 nL loop (1 ng absolute amount). The flow rate of ACN was set at 400 nL·min^–1^ with a 30 μm i.d. capillary that was 2 cm inside the VMC. The IS was analyzed in the same conditions. Once the LEI was optimized, the MOI performance was adjusted. For this purpose, the following parameters were considered: (1) the flow isolated volume inside the MOI, which must be the smallest possible, and (2) the choice of capillaries based on the internal diameter and length, which must not create excessive pressure on the system and must ensure the least-possible volume for a rapid transfer of the analytes to the MS. As shown in Figure 2, MOI isolation was ensured by switching the bypass valve (valve 2). In position A, the flow rate goes from valve 1 to the MOI (port 4 valve 2). This port communicates with port , which corresponds to the MOI entrance. Once the MOI chamber is filled, the flow goes to port 6 and then through port 1 directly to LEI-MS/MS. When valve 2 is switched to position B (bypass), the flow rate goes directly from port 4 to waste (port 3). In this position, ACN containing the IS solution is isolated in the MOI chamber. The MOI chamber i.d. must be the smallest possible but large enough to allow the fiber insertion and promote its correct desorption (without damaging the coating surface). The solvent inside the chamber acts as a lubricant, facilitating the insertion of the fiber without any effort. A 500 μm i.d. and 1/16 o.d. PEEK capillary was selected for the chamber assembly. Considering the fiber coating length (1 cm) and the volume surrounding the fiber inside the 500 μm i.d. chamber, the ACN volume involved in desorbing the sorbent surface was calculated as 2.5 μL. The length of the PEEK capillary was the shortest possible according to the standard 1/16 connection’s sizes. A 5 cm piece was thus selected. The other capillaries completing the MOI structure were all PEEK silica of different internal diameters and lengths, as shown in Figure 1. The capillaries carrying the IS solution from the pump to the MOI (through valves 1 and 2) had a 75 μm i.d. to avoid overpressuring. The other capillaries connecting the MOI to the MS through valve 2 were selected to reduce dead volumes to a minimum, ensuring the fastest sample transfer to MS. The optimal configuration was obtained with the capillaries reported in Figure 1.

Once the MOI system was configured correctly, system robustness was evaluated. A 2.5 μL solution of fentanyl in ACN at 2 mg·L^–1^ was directly introduced in the MOI chamber using a manual syringe. This 2.5 μL solution simulated the flow-isolated volume after SPME fiber desorption. The analysis procedure was the same as that described in step 3 of the paragraph MOI Description, Modification, And Operation. Different flow rates at 100% ACN between 200 and 800 nL·min^–1^ were tested. It was observed that increasing the flow rate implies not only a smoother signal but also a decrease in sensitivity. Therefore, it is necessary to find an equilibrium between a smooth peak shape and the sensitivity, and 400 nL·min^–1^ was selected as the optimal flow rate. The intraday relative standard deviation (RSD, *n* = 4) was 8.2%. Fentanyl-D5 was tested in the same conditions as those for fentanyl, obtaining satisfactory results. The RSD obtained for the analysis of 2.5 μL of fentanyl-D5 at 2 mg·L^–1^ was 6.3% (*n* = 4). During normal operations, the IS solution was provided by the 100 μL injection loop in valve 1, which was a sufficiently large volume for a high number of experiments at 400 nL·min^–1^.

### Optimization of the DI-SPME MOI-LEI-MS/MS Method

Several factors were investigated to increase the extraction efficiencies during the DI-SPME procedure. These factors include the desorption solvent, the extraction and desorption time, the pH, and the percentage of organic solvent in the samples. For this assessment, the initial conditions were set as follows: 3 mL of a fentanyl (200 μg·L^–1^) aqueous standard solution with 0.3% in MeOH (v/v), an extraction time of 30 min, and a desorption time of 1 min. Each parameter was calculated in triplicate to obtain the corresponding error. The extraction efficiency of fentanyl was calculated based on peak areas of the *Q* transition (indicated in the Experimental Section and Table S1). All parameters were evaluated in deionized water and fentanyl-free urine.

### Desorption Solvent

The first parameter tested was the desorption solvent in the conditions described in the above paragraph. ACN and MeOH were taken into account due to their compatibility with the nano-LC system and a high capacity to dissolve fentanyl. In Figure S1, the desorption efficiency of ACN and MeOH are reported in terms of peak areas. These data show that ACN is more efficient than MeOH as a desorption solvent. The desorption solvents were evaluated in water samples, and the results were extrapolated for urine and plasma.

### Effect of pH

**Figure 3:**
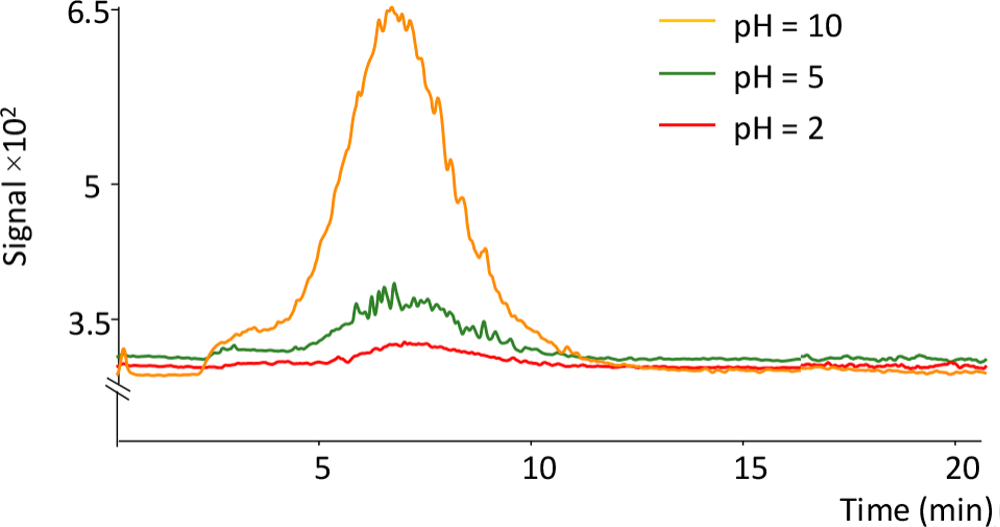
Influence of pH in the determination of fentanyl in urine samples.

The pH of the aqueous sample affects analytes carrying basic or acidic groups, varying their dissociation equilibria. It is essential to select an appropriate pH to ensure that fentanyl is in neutral form before extraction. The p*K*_a_ of fentanyl is 8.4 at 25 °C; therefore, a basic aqueous solution should increase the extraction efficiency. Aqueous standard solutions of fentanyl at 100 μg·L^–1^ were prepared at pH 2, 5, 7, and 10 and extracted as previously described. H_2_SO_4_ (0.1 mol·L^–1^) and NaOH (5 mol·L^–1^) were used to adjust the pH levels of the samples. Higher pHs were not considered in order to avoid fiber damage. In aqueous solutions, the pH does not significantly influence the extraction efficiency. However, considerable differences were observed in urine. In Figure 3, the overlapped signals of spiked urine samples at different pHs are shown. It was observed that the highest signal was registered at pH 10. This result may be explained by the fact that urine proteins precipitate at basic pHs.^42−44^ Fentanyl is likely bound to urine proteins and then released at a basic pH. Therefore, this parameter has a significant relevance in the determination of fentanyl in biological samples. It is also essential to consider that fentanyl is present in its neutral form in aqueous solutions at basic pHs and is easily adsorbed on a C18 fiber.

### Effect of Organic Solvents in Extraction Media

In DI-SPME, the presence of an organic solvent in the matrix can enhance the analyte partitioning into the fiber coating, increasing the solubility of the analytes in the sample. The lowest amount of organic solvent in the matrix should be used to avoid competition with the stationary phase of the fiber. MeOH was used in this evaluation because it was the solvent the commercial standard was dissolved in. In aqueous samples (Figure S2A), MeOH was tested at the following percentages (v/v): 0.5%, 1%, 3%, 5%, and 8% (including the spiked amount). No significant differences in peak areas were observed at lower percentages of MeOH. Therefore, the percentage of MeOH in aqueous samples was set at 0.5% (v/v) to use the lowest possible amount of organic solvent. In urine (Figure S2B), this effect was monitored by measuring the peak areas of fentanyl at the following MeOH percentages (v/v): 0.5%, 1%, 5%, 10%, and 20%. In the case of a complex matrix, a higher percentage of the organic solvent can be used to favor the adsorption on SPME in by either promoting a partial precipitation of the other components (thus, avoiding interferences) or increasing their solubility to avoid competition with the target analyte during the extraction process. The highest extraction efficiencies in terms of the peak areas was obtained with 5% and 20% MeOH (v/v). However, 5% MeOH was preferred due to the lower relative standard deviation (RSD). At higher percentages of MeOH, the measurement’s instability increases. This is due to partition equilibria within the analyte–organic solvent and analyte–solid sorbent.^45^

### Influence of the Stirring Speed

Sample agitation is often carried out with a small stirring bar to decrease the equilibration time. Different magnetic stirring speeds were tested, namely no stirring, 300, 500, 700, and 1000 rpm, and the results were evaluated in terms of peak areas. No stirring or low stirring speeds imply a longer time for analyte partitioning. On the other hand, stirring too fast may cause the opposite effect, and the analytes return to the aqueous phase. As demonstrated in Figure S3, the best results were obtained at 700 rpm in both matrices.

### Optimization of Extraction and Desorption Times

The extraction time profiles were obtained at room temperature for 20, 30, 45, 60, 90, and 120 min. The MOI desorption time was 1 min in all cases. The optimal extraction efficiency of fentanyl in water is 60 min, whereas that in urine is 90 min, as demonstrated in Figure S4A and B. However, an extraction time of 30 min ensures a satisfactory result in a much shorter time with a limited signal decrease. The desorption time was also evaluated at the optimal extraction time to guarantee the absence of carry-over and maximize the efficiency. Thus, at room temperature with 700 rpm stirring and 60 and 30 min of extraction time for water and urine, respectively, the tested desorption times were 0.5, 1, 2, and 5 min. From Figure S4C and D it is evident that 1 min ensured the highest desorption efficiency for both matrices, with no memory effects as tested in further blank analyses.

**Figure 4:**
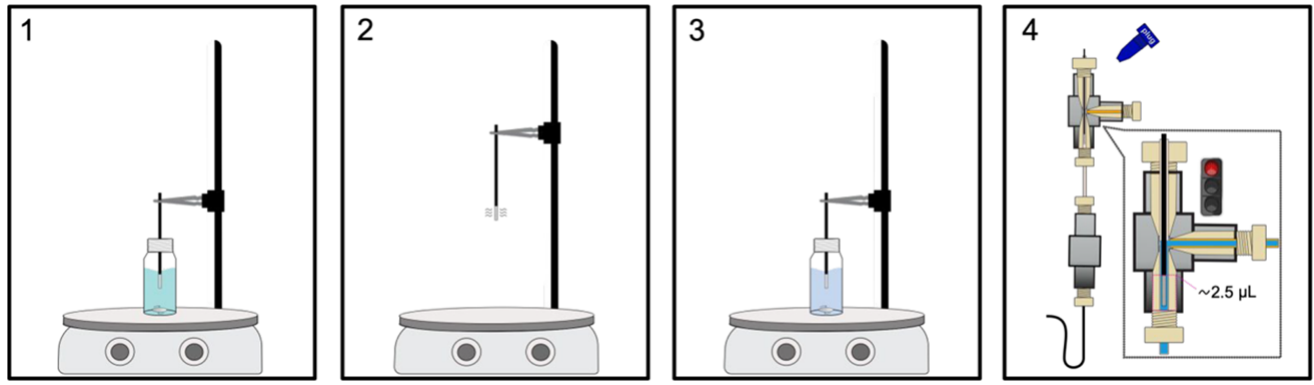
DI-SPME workflow.

In summary, the optimal conditions include direct immersion of the Bio-SPME fiber in 3 mL of the sample at pH 7 and 0.5% MeOH (v/v) for water samples and pH 10 and 5% MeOH (v/v) for urine samples, extraction at 700 rpm and room temperature for 60 min in water and 30 min in urine, and a desorption time in MOI of 1 min in all cases. The workflow is shown in Figure 41–4.

### DI-SPME Method Performance

The performance of the DI-SPME method using a Bio-C18 coating was evaluated on aqueous standards and urine, applying to each matrix the optimal extraction conditions detailed above. Table 1 shows method validation data for the two matrices. A seven-point calibration curve was determined in triplicate for both matrices using the following concentrations: 10, 50, 100, 200, 500, 750, and 1000 μg·L^–1^. Linearity was excellent in both matrices, with a determination coefficient value (*R*^2^) of 0.9996 in water and 0.9990 in urine. Limits of detection (LODs) were calculated as 3× the signal-to-noise ratio and were 3.7 μg·L^–1^ in water and 4.1 μg·L^–1^ in urine. Limits of quantitation (LOQs) were calculated as 10× the signal-to-noise ratio and were 12.3 μg·L^–1^ for water and 13.7 μg·L^–1^ for urine. LODs and LOQs were calculated using the *q* transition.

**Table 1:** Method Validation Data

									RSD (%) at 200 μg·L^–1^	
matrix	linearity range (μg·L^–1^)	levels	*R*^2^	*S*_y/x_	slope ± SD	intercept ± SD	LOD (μg·L^–1^)	LOQ (μg·L^–1^)	interday^a^	intraday^b^
water	12.3–1000	6	0.9996	3957	162 ± 4	–34 ± 164	3.7	12.3	10.6	9.2
urine	13.7–1000	6	0.9990	1048	117 ± 1	5190 ± 573	4.1	13.7	14.5	6.6

a Interday studies (*n* = 3 each day for 3 nonconsecutive days).

b Intraday studies (*n* = 4).

Precision was evaluated as the relative standard deviation (RSD %) by performing intraday (*n* = 4) and interday repeatability measurements (*n* = 3 each day for three nonconsecutive days) using a 200 μg·L^–1^ standard solution. Intra- and interday RSD values were 10.6 and 9.2%, respectively, for water and 14.5 and 16.6%, respectively, for urine, thus demonstrating good repeatability.

### Matrix Effects Evaluation

**Figure 5:**
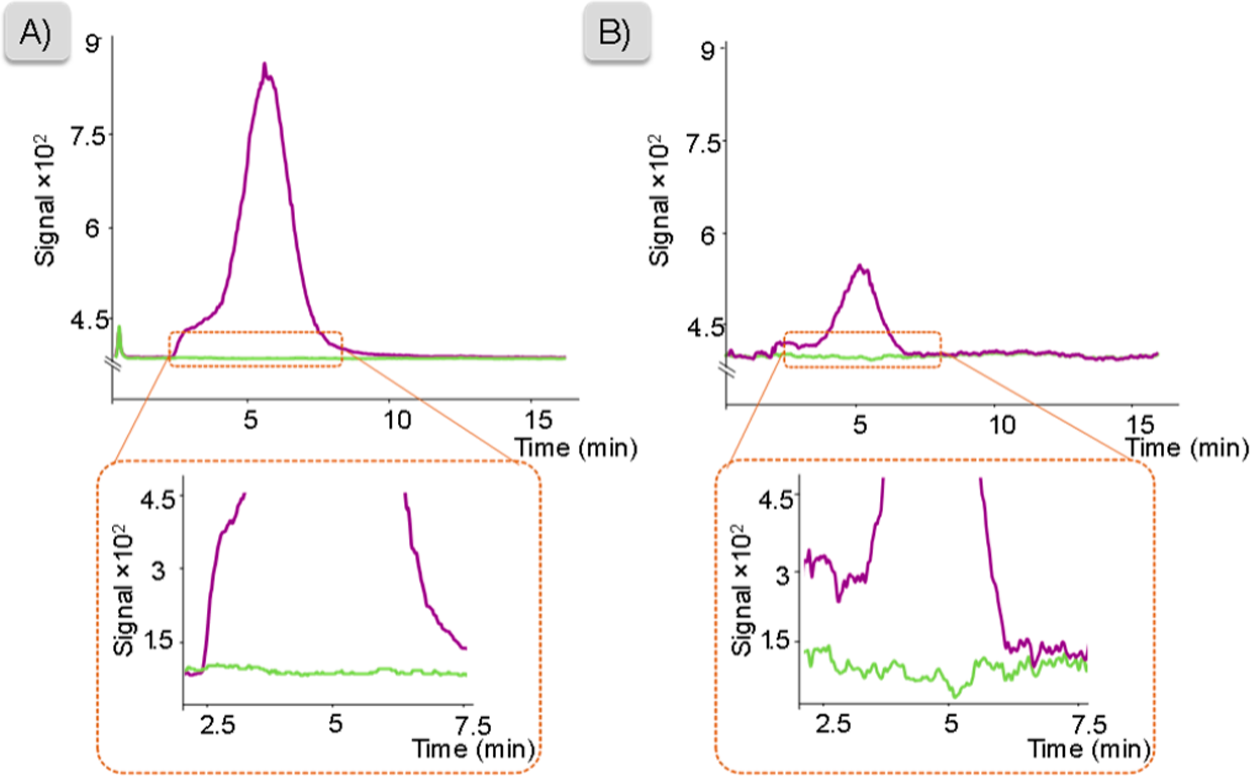
Evaluation of ME using a continuous flow of fentanyl-D5 as the IS with (A) urine and (B) plasma diluted 1:1 (v/v) in water. The purple line is fentanyl (200 μg·L^–1^), and the green line is fentanyl-D5 200 (μg·L^–1^).

Matrix-dependent signal suppression or enhancement (ME) represent a significant limitation in LC-MS quantitative analysis, especially when chromatography is insufficient to separate the analytes from possible interfering coeluted compounds. In the proposed method, the MOI allows the quick desorption of the fiber and the subsequent introduction of the sample into the MS without chromatographic separation; therefore, the coeluting matrix components and analytes coexist in the ion source. LEI, due to gas-phase ionization, is well-known for not being affected by ME, which is from liquid ionization-based methods, ESI in particular, where coeluted compounds compete for the available charges. One goal of this work is the evaluation of ME in the MOI-LEI-MS/MS system using two different matrices, urine and plasma. ME can be estimated following different approaches.^46,47^ Because SPME performance and properties have already been extensively evaluated and discussed elsewhere, the focus was placed on the contribution of ME coming from LEI-MS/MS alone. As described in the Experimental Section, another six-port valve (valve 1) with a 100 μL loop was placed between the nano-LC pump and valve 2 (Figure 1). The loop was filled with 100 μL of fentanyl-D5 at 2 mg·L^–1^ as the IS. Thanks to the configuration described, a constant concentration of IS in ACN was admitted in the system as a desorption solution. In this way, once fentanyl was desorbed in MOI, the nano-LC pushed it, together with the IS, directly to LEI-MS/MS. Different concentrations of fentanyl were analyzed in triplicate, and the IS signal was monitored simultaneously to evaluate the degree of ME caused by the MS ionization source alone. These experiments are very similar in concept to the postcolumn infusion method in which a constant concentration of an IS is added to the eluate after chromatographic separation, generating a constant and flat IS signal in absence of ME. In our case, suppression or enhancement of the IS signal in the presence of ME should be observed corresponding to the fentanyl peak. For this test, the two matrices considered were urine and plasma (diluted 1:1 (v/v) in water), both of which were spiked with fentanyl at 200 μg·L^–1^. As shown in Figure 5A (urine) and B (plasma), IS signals did not show significant variations during fentanyl elution for both matrices, proving that no ME can be ascribed to LEI-MS/MS detection. However, it can be noticed that the fentanyl signal in plasma is less intense than that in urine. This difference cannot be counted as ion suppression originating in the ion source, as demonstrated, and can instead be attributed to the extraction step due to the high complexity of plasma. For the same reason, the IS signal is noisier in plasma than in urine, without showing any ME-related variations during fentanyl elution.

## Conclusions

The present research work proposes a novel interface for direct Bio-SPME fiber desorption in combination with LEI-MS/MS detection. This system has been used for the determination of the amount of fentanyl in urine and plasma. The system, thanks to a revisited MOI, was adapted to work at nanoscale flow rates, offering the ME-free and accurate quantitation of the analyte in two different biological matrices. The new configuration permits not only desorption of the analyte from the fiber but also a fast analysis under a constant flow of IS (fentanyl-D5) for maximum accuracy. LEI represents the ideal pairing for the type of compounds compatible with a C18 Bio-SPME fiber, and other applications are under investigation. This proof-of-concept demonstrates the successful coupling of SPME and LEI. However, the following two critical points need to be addressed: the speed of the analysis and LODs. The first one mainly depends on the extraction time, whereas the second is related to the very low flow rate that causes broad signals. Our group is actively working on optimizing the extraction procedure and MOI internal volumes using custom-made components for a faster sample transfer and reduced analysis time.

## Supporting Information Available

MS parameters for data acquisition, evaluation of different desorption solvents, influence of the percentage of the organic solvent in different matrices, influence of the stirring speed, and extraction and desorption time studies (PDF)
