# Supplementary material for: Direct Coupling of Bio-SPME to Liquid Electron Ionization-MS/MS via a Modified Microfluidic Open Interface
Source: J Am Soc Mass Spectrom. 2020 Nov 20;32(1):262–9. doi: 10.1021/jasms.0c00303 (PMC8016190; doi:10.1021/jasms.0c00303)
Supplement: Supplementary file 1 — js0c00303_si_001.pdf [file js0c00303_si_001.pdf]

SUPPORTING INFORMATION

Direct Coupling of Bio-SPME to Liquid Electron Ionization-MS/MS via a Modified Microfluidic Open Interface

Priscilla Rocío-Bautista[1,2], Giorgio Famiglini[2], Veronica Termopoli[2], Pierangela Palma[2,4], Emir Nazdrajić[3], Janusz Pawliszyn[3], Achille Cappiello[2,4*]

[1]*Department of Chemistry, Life Sciences and Environmental Sustainability, University of Parma, 43121, Parma, Italy*
[2]*Department of Pure and Applied Sciences, University of Urbino, 61029, Urbino, Italy*
[3]*Department of Chemistry, University of Waterloo, Waterloo, ON, N2L 3G1, Canada*
[4]*Chemistry Department, Vancouver Island University VIU, Nanaimo, BC, V9R5S5 Canada*

*Corresponding author: Tel. +39 0722303344; e-mail: achille.cappiello@uniurb.it
Orcid ID: 0000-0001-6149-4305

Table of Contents

Table S1: MS parameters for data acquisition.

Figure S1: Evaluation of different desorption solvents.

Figure S2: Influence of the percentage of organic solvent in different matrices.

Figure S3: Influence of the stirring speed.

Figure S4: Extraction and desorption time optimization studies.

Table S1. MS parameters for data acquisition

Compound	Structure	Quantifier transition	Collision energy (eV)	Qualifier transition	Collision energy (eV)
Fentanyl	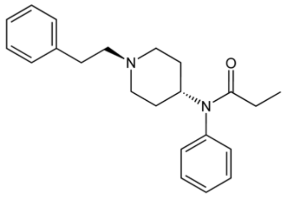	245 – 189	10	245 – 146	5
Fentanyl-D5	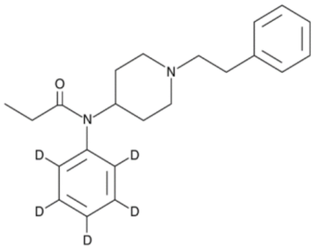	250 – 194	10	250 – 151	10

He flow rate: 1 mL·min^{-1}

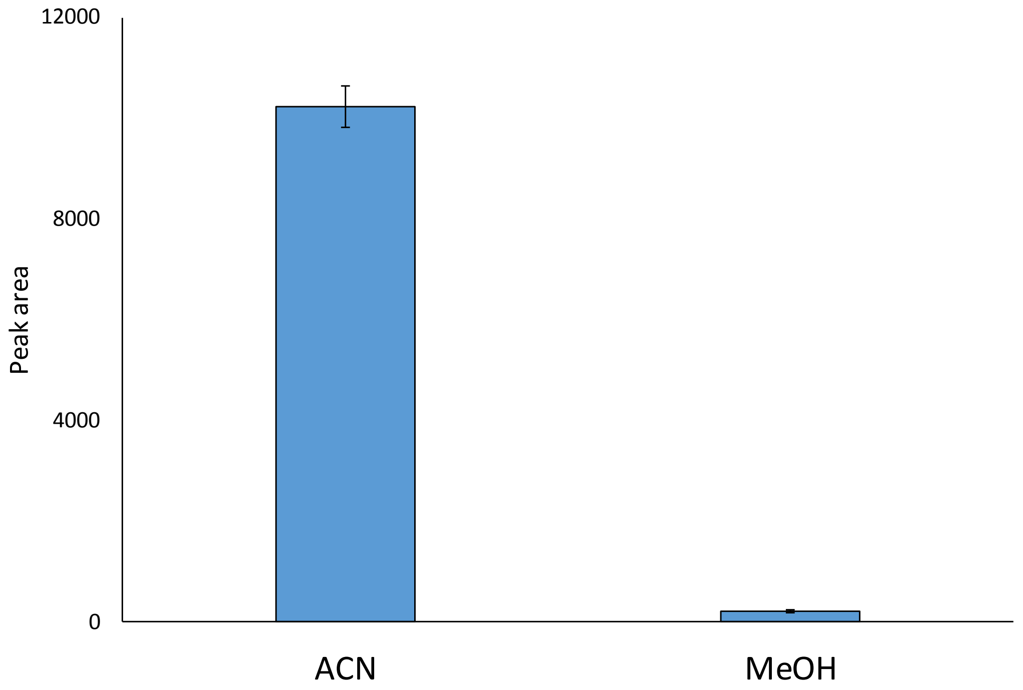

Figure S1. Evaluation of different desorption solvents. The extraction was performed with a 200 µg·L^{-1} solution of fentanyl in water, with 30 min extraction time, and 1 min desorption time. (n=3).

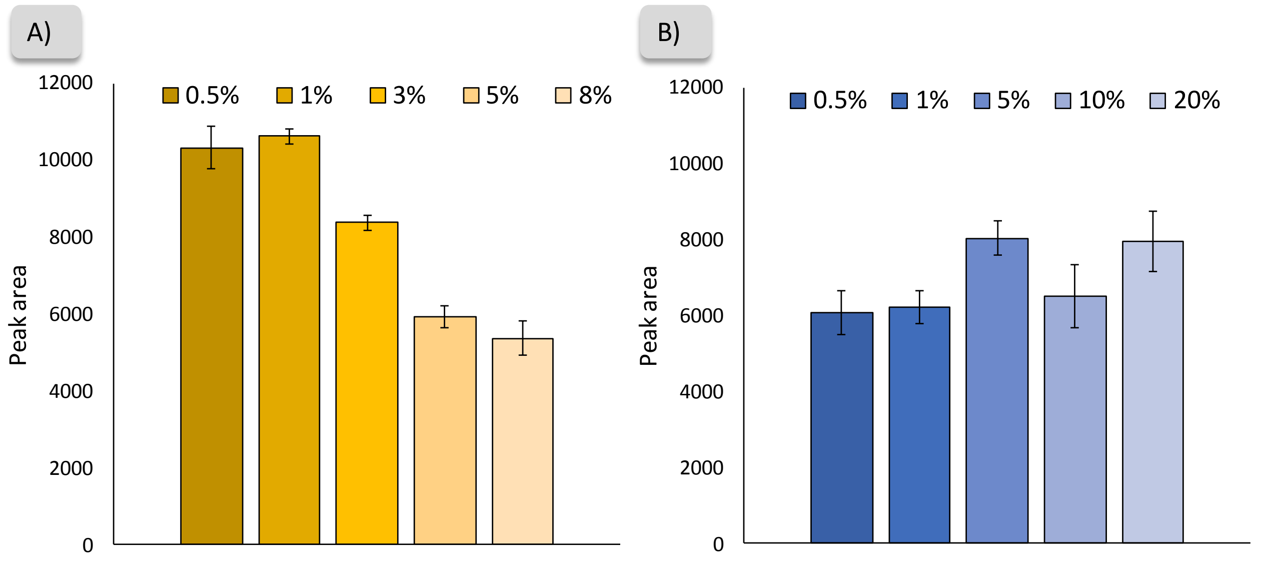

Figure S2. Influence of the percentage of organic solvent in different matrices. A) Aqueous standard solution. B) Urine. (n=3).

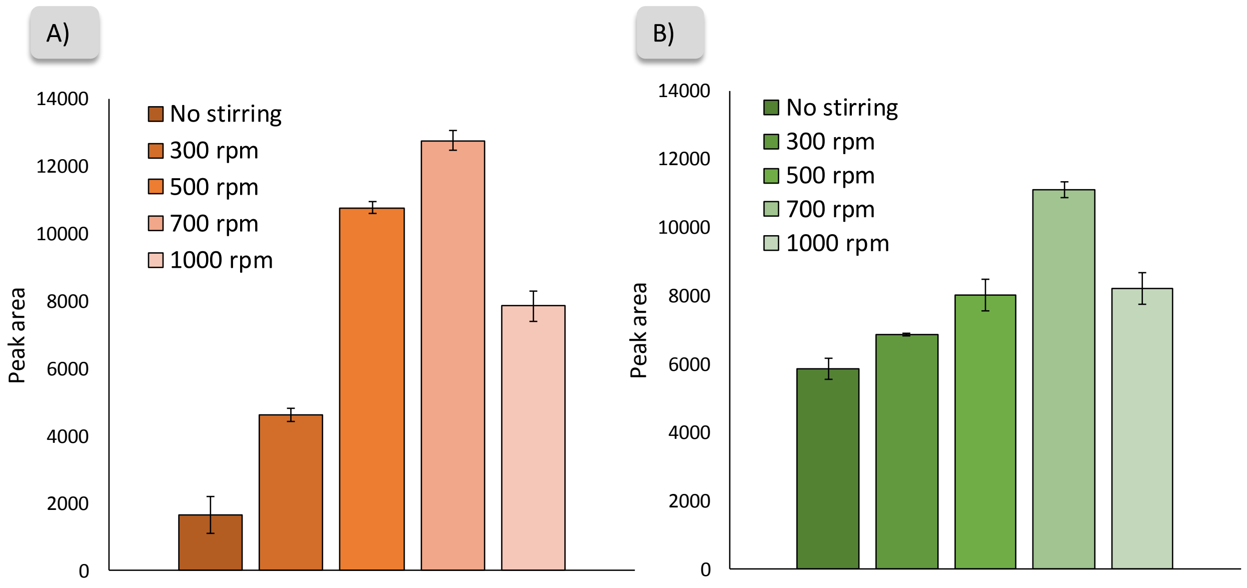

Figure S3. Influence of the stirring speed in A) aqueous standard solution and B) urine. (n=3).

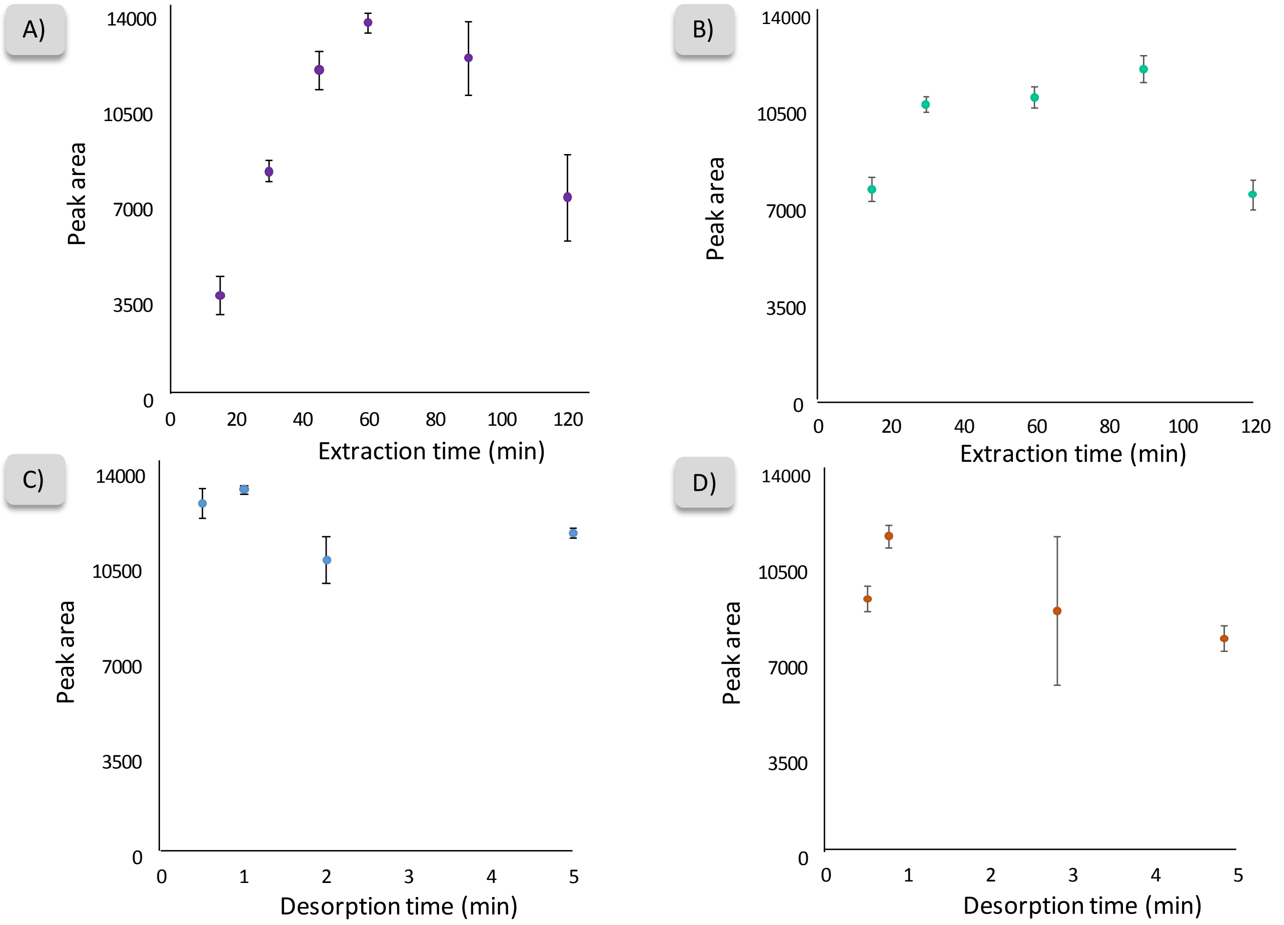

Figure S4. Extraction and desorption time optimization studies. Extraction time profile were obtained using a desorption time of 1 min (n=3). A) extraction time profile for water sample; B) extraction time profile for urine sample; C) desorption time for water sample; D) desorption time profile for urine sample.
